# Annular elastolytic giant cell granuloma mimicking cutaneous sarcoidosis

**DOI:** 10.1002/ccr3.5709

**Published:** 2022-04-08

**Authors:** Mariem Daldoul, Mouna Korbi, Hayet Akkari, Badreddine Sriha, Hichem Belhadjali, Jameleddine Zili

**Affiliations:** ^1^ Department of Dermatology Fattouma Bourguiba University Hospital of Monastir Monastir Tunisia; ^2^ Department of Pathology Farhat Hached University Hospital of Sousse Sousse Tunisia

**Keywords:** dermatology, endocrinology and metabolic disorder, granuloma, sacroidosis

## Abstract

Annular elastolytic giant cell granuloma (AEGCG) is a benign skin disorder, with, unknown cause. It appears as erythematous papules or annular plaques. Few challenging cases of AEGCG have been reported in the literature. We describe a rare clinical presentation of AEGCG mimicking cutaneous sarcoidosis.

## Graphical abstract

Annular elastolytic giant cell granuloma is a rare inflammatory disorder with an unknown cause. The clinical presentation is annular erythematous plaques on sun exposed areas. Histopathlogy shows multinucleate giant cells and elastin degeneration.

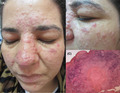

Annular elastolytic giant cell granuloma (AEGCG) is a rare inflammatory disorder with an unknown cause.^1^ It was first described in 1979 by Hanke et al^1^ as erythematous papules or annular plaques with raised defined borders and atrophic center. We describe a rare clinical presentation of AEGCG mimicking cutaneous sarcoidosis.

A 38‐year‐old woman presented with a 3‐month history of monomorphic eruption on the face. Dermatological examination revealed multiple erythematous and non‐itchy papules of 2–3 mm diameter, distributed symmetrically on centro‐facial areas (Figure 1A,B).

**FIGURE 1:**
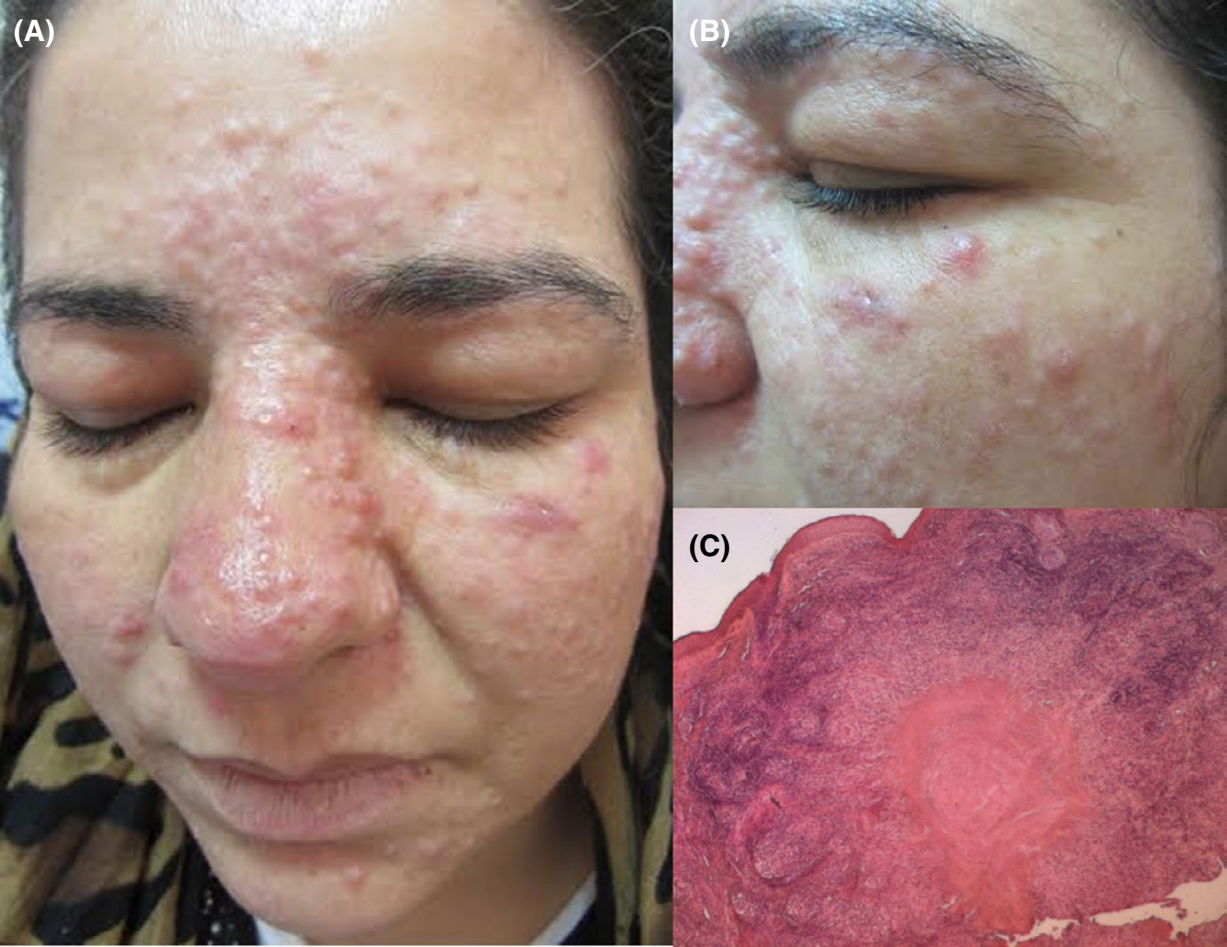
(A) Numerous erythematous and non‐itchy papules, of 2–3 mm diameter (B) distributed symmetrically on the face (C) palisading granulomatous inflammation and foci of degenerative collagen

All biological explorations were within normal levels. Histopathological examination of skin biopsy showed foci of degenerative collagen and palisading granulomatous inflammation (Figure 1C).

Based on clinical and histopathological findings, the diagnosis of AEGCG was made. The diagnosis of AEGCG can be challenging especially when it is clinically presented by papular lesions. It can be confused with granuloma annulare (GA) or sarcoidosis. Some authors consider that AEGCG is a GA occurring in the actinic elastosis areas. However, histological finding suggests that AEGCG and GA are two separate entities. In fact, skin biopsy of AEGCG shows granulomatous inflammation with giant multinucleated cells, elastolysis, and elastophagocytosis.^2^ It can be differentiated from GA by the absence of necrobiosis and palisading granuloma.^2^

## CONFLICT OF INTEREST

None.

## AUTHOR CONTRIBUTIONS

All the authors contributed to the writing of the manuscript.

## CONSENT

Written informed consent was obtained from the patient to publish this report in accordance with the journal's patient consent policy.

## Data Availability

Data sharing not applicable—no new data generated.
